# The Gut Microbiome of the Asiatic Toad ( *Bufo gargarizans* ) Reflects Environmental Changes and Human Activities

**DOI:** 10.1002/ece3.71394

**Published:** 2025-05-07

**Authors:** Xuena Kang, Meiying Shao, Jiyang Jiang, Lewei He, Yunwei Lu, Jiarong Song, Jue Xu, Zhenxin Fan

**Affiliations:** ^1^ Key Laboratory of Bioresources and Ecoenvironment, Ministry of Education, College of Life Sciences Sichuan University Chengdu China; ^2^ Sichuan Key Laboratory of Conservation Biology on Endangered Wildlife, College of Life Sciences Sichuan University Chengdu China; ^3^ West China School of Public Health and West China Fourth Hospital Sichuan University Chengdu China

**Keywords:** *Bufo gargarizans*, *Citrobacter*, human activities, metagenome‐assembled genomes, metagenomic

## Abstract

Amphibians are extremely sensitive to environmental changes, and their gut microbiome may have different responses to environmental changes. Here, metagenomic sequencing was used to investigate the intestinal microbiota of the Asiatic toad ( *Bufo gargarizans* ) from three different habitats (city areas, transition areas, and wild areas) of Sichuan Province, China. The results showed that Proteobacteria, Firmicutes, and Fusobacteria were the main bacteria in the gut of *B. gargarizans* . There were significant differences in the composition and function of the gut microbiome among the samples from the three different habitats. Enterobacteriaceae showed significant changes in the three habitats and occupied a high relative abundance in the city areas, especially for *Citrobacter*. Especially, antibiotic resistance genes (ARGs) and virulence factors (VFs) were significantly increased in city areas. We performed de novo assembly of the metagenome‐assembled genomes (MAGs). In total, 322 nonredundant MAGs were reconstructed, 304 of which might be potential novel genomes*.* Among the 13 species‐level genome bins (SGBs) belonging to Enterobacteriaceae, the one belonging to *Citrobacter portucalensis* annotated the most types of ARGs and VFs. Phylogenetic and functional analyses of the assembled *C. portucalensis* MAG and public genome data were carried out, suggesting that it may play a potential role in intestinal diseases in amphibians. Our study revealed the differences in the gut microbiome of *B. gargarizans* across different habitats and suggests that amphibian intestinal microbiota could serve as environmental indicators to reflect environmental changes and human activities. The reconstructed MAGs expanded our understanding of the gut microbiota in amphibians, which may serve as a substantial reservoir for microbiome resources.

## Graphical abstract

Our study provided valuable insights into the characteristics of the intestinal microbiota in different habitats of *B. gargarizans* by using metagenomic sequencing. Our work suggested that the intestinal microbiota of *B. gargarizans* could be used as one of the environmental indicators to reflect environmental pollution and changes. Furthermore, the accumulation of Enterobacteriaceae, particularly Citrobacter, in the intestines of *B. gargarizans* in city areas is noteworthy.

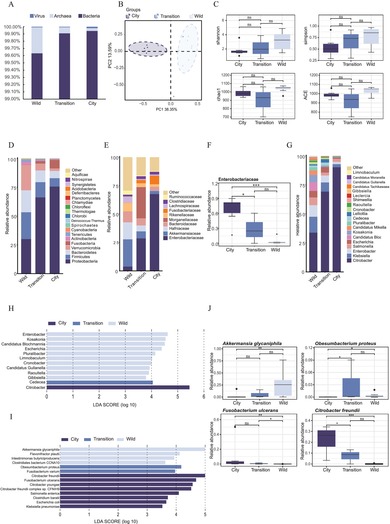

## 1 Introduction

Intestinal microbiota plays an important role in host function, such as enhancing the intestinal epithelial barrier, maintaining immune balance, modulating immune responses, and inhibiting pathogen colonization (Adak and Khan 2019; Fan and Pedersen 2021; Wang et al. 2023). Amphibian guts host a diverse microbial community (Colombo et al. 2015), which plays an important role in the health and function of these animals (Long et al. 2020; Wiebler et al. 2018). The composition and function of the intestinal microbiota interact with the host's diet, environment, health status, developmental stage, and more (Brunetti et al. 2023; Fontaine et al. 2021; Trevelline and Kohl 2022; Yatsunenko et al. 2012). Amphibian communities are extremely sensitive to environmental changes compared to other vertebrate groups (Hopkins 2007), and habitat changes have a significant impact on their species composition and diversity (Decena et al. 2020). Their intestinal microbiota also have different responses to environmental changes and are habitat‐specific (Bletz et al. 2016). Therefore, the living environment of amphibians may be the main filter for their gut microbial communities.

Among amphibians, the Asiatic toad ( *Bufo gargarizans* ) is a widespread and abundant species found in China, Russia, North Korea, South Korea, and Japan (Hu et al. 2007). Its skin is permeable with small holes, lacking protective structures such as an amniotic membrane or shell from the onset of fertilized egg development, making *B. gargarizans* highly sensitive to environmental changes. Therefore, it is also used as an indicator for monitoring environmental pollution (Bo et al. 2018; Li et al. 2018; Zheng et al. 2021). Consequently, its intestinal microbiota can also be used as a monitoring indicator of its living environment to reflect environmental changes and environmental pollution (Liu et al. 2022; Lv et al. 2023; Xie et al. 2020; Yao et al. 2019; Zheng et al. 2020).

Some studies have used advanced next‐generation sequencing to explore the intestinal microbiota characteristics of *B. gargarizans* , but they are usually based on 16S rRNA sequencing analysis (Chai et al. 2018; Chai et al. 2022; Song et al. 2023; Xu et al. 2020). However, The accuracy of species‐level annotation and gene prediction using 16S rRNA sequencing is limited (Ranjan et al. 2016), and our understanding of the structure and characteristics of the intestinal microbiota in *B. gargarizans* , as well as their responses to environmental changes, remains limited. In contrast, metagenomic sequencing can provide a more comprehensive and in‐depth characterization of the complexity of the microbiome (Laudadio et al. 2018), and can be achieved by obtaining nearly complete metagenomic assembled genomes (MAGs) on a large scale to better reveal the composition and function of the intestinal microbiota (Li et al. 2023).

In this study, metagenomic sequencing was used to conduct a comprehensive study of the intestinal microbiota of *B. gargarizans* . We collected samples from three habitat types (city areas, transition areas, and wild areas) to analyze the differences in composition and function of the intestinal microbiota among individuals in habitats with different levels of human activity. In addition, MAGs in the intestinal microbiota were assembled to further study the function of the intestinal microbiota of *B. gargarizans* . Specifically, *Citrobacter portucalensis* may play a potential role in animal diseases (Sellera et al. 2023; Thomas et al. 2020). Therefore, phylogenetic and functional analyses of the assembled *C. portucalensis* were carried out to provide references for disease research in amphibians. This study aims to deepen our understanding of the composition of gut microbiota and the influence of human activities on amphibians. Ultimately, our findings serve as a warning for amphibian protection and environmental management.

## 2 Materials and Methods

### 2.1 Sample Collection

We collected 16 intestinal samples from *B. gargarizans* in city, transition, and wild areas of Sichuan Province, China. Specifically, samples from forested areas in Kangding City (*n* = 3) and Tibetan Autonomous County of Muli (*n* = 4) were defined as the wild group, Dujiangyan City (*n* = 3) as the transition group, and Shuangliu district (*n* = 3) and Jinjiang district (*n* = 3) in Chengdu City as the city group (Table S1 and Figure S1), based on the density of population and human activity levels. The Wild was defined as sites located approximately 5000 m away from human activity and within areas with a population density of fewer than 9 persons/km^2^. The Transition was defined as a site within 300 m of human activity and in areas with a population density of 426 persons/km^2^. The city was defined as sites within 100 m of human activity and in areas with a population density exceeding 910 persons/km^2^. All experimental toads were healthy and energetic. They were transported to the laboratory immediately after collection. The toads were placed in wide‐mouth bottles containing cotton balls soaked in ether and allowed to sit for 10 min before being euthanized by pithing the brain and spinal cord. Each toad was wiped three times with 75% alcohol. After dissection, the digestive tract was carefully separated from the body, and sterile tweezers were used to squeeze and collect the contents (excluding the stomach). The digestive tract was then rinsed twice with sterile phosphate‐buffered saline (PBS) and collected. A new pair of sterile tweezers was used for each toad to avoid contamination. The intestinal contents were collected and frozen at –80°C for DNA extraction. This study was approved by the Ethics Committee of the College of Life Sciences, Sichuan University (No. 20210309009).

### 2.2 DNA Extraction and Metagenomic Processing

We extracted the total DNA according to the TIANNamp Stool DNA Kit (Tiangen Biotech Co. Ltd., China) instructions and measured DNA concentration and quality with a NanoDrop. All of the above operations were performed in a sterile environment; the concentration of the final library was > 5 ng/μL in a volume of 50 μL. The qualified DNA library was sequenced using an Illumina NovoSeq 6000 platform (Novogene Co. Ltd., China) with a paired‐end sequencing length of 150 bp. The adapters and low‐quality reads were filtered by Trimmomatic (v0.39) (Bolger et al. 2014), while hosts' sequences (including human contamination) were removed by Bowtie2 (v2.4.5) (Langmead and Salzberg 2012) based on its NCBI reference genome (https://www.ncbi.nlm.nih.gov/genome/8043). All raw data have been submitted to the China National GeneBank DataBase (https://db.cngb.org/) with the accession number CNP0005874.

### 2.3 Identification of Taxa and Function Prediction in Metagenomes

Kraken2 (v2.1.2) was used for species taxonomic annotation (Wood et al. 2019), and the abundance of taxa was normalized to relative abundance. All taxa were retained for subsequent analysis. Assembly analysis was performed by MEGAHIT (v1.2.9) (Li et al. 2015) with the option “—min‐contig‐len 300”. In addition, CD‐HIT (v4.8.1) (Fu et al. 2012) was used with default parameters (identity 95%; coverage 90%) to cluster and remove redundant sequences, thus obtaining nonredundant gene set sequences. The quantification of these nonredundant genes in each sample was performed using Salmon (v1.3.0) (Patro et al. 2017). The gene prediction results were obtained by Prodigal (v3.0.2) (Hyatt et al. 2010). The abundance of gene families and microbial metabolic pathways was assessed by HUMAnN3 using MetaCyc and UniRef90 EC filtering databases (Franzosa et al. 2018; Suzek et al. 2007). Antibiotic resistance genes (ARGs) were quantified using RGI (v6.0.3) with the comprehensive antibiotic resistance database (CARD v3.2.9) (Alcock et al. 2023). Virulence factors were predicted using diamond (v2.1.9) with the Virulence factor database (VFDB) (Chen et al. 2005). The results were standardized using transcripts per million (TPM). Default parameters were used for software without specified parameters.

### 2.4 Reconstruction of Metagenome‐Assembled Genomes (MAGs)

The contigs larger than 300 bp assembled by MEGAHIT (v1.2.9) were processed, and Bowtie2 (v2.4.5) was used to compare the assembled data with metagenomic short sequences. The sequence alignment/map (SAM) result files were converted into binary alignment/map (BAM) files using SAMtools (v0.1.19) and sorted (Li et al. 2009). Metagenomic binning was performed using MetaBAT2 (v2.17) (Kang et al. 2019).

Raw bins were dereplicated at a default threshold of 99% average nucleotide identity (ANI) using dRep (Olm et al. 2017) with the option dereplicate_wf ‐comp 70‐con 10‐sa 0.99‐nc 0.3‐S_algorithm fastANI. A set of nonredundant bins with completeness > 70% and contamination < 10% was obtained as the final result to analyze. Based on these results, bins were also dereplicated using dRep at a threshold of 95% ANI to obtain species‐level genome bins (SGBs). The completeness and contamination of all bins were evaluated by CheckM (v1.1.6) (Parks et al. 2015).

### 2.5 Prediction of Taxonomy Label of MAGs

The putative taxonomy label of MAGs was predicted using the Genome Taxonomy Database Toolkit (GTDB‐Tk) (Chaumeil et al. 2019) with the “classify_wf” functional module based on Genome Taxonomy Database (GTDB) taxonomy (Parks et al. 2020). Phylogenetic trees were built with the “de novo wf” function and converted into Interactive Tree of Life (iTOL, https://itol.embl.de/) readable format using the “convert to itol” function for final visualization and rendering. The functional module of “annotate_bins” was used to annotate bins in MetaWRAP (v1.3). The genes were aligned to eggNOG database (Huerta‐Cepas et al. 2019) using eggNOG‐Mapper (v2.1.12) (Cantalapiedra et al. 2021). CAZymes genes were identified using dbCAN3 (v3.0) (Zheng et al. 2023) based on CAZy database (Drula et al. 2022). KEGG orthologs (KOs) were annotated using KofamKOALA (vKEGG release 110.0) (Aramaki et al. 2020). Alongside data from the present study, public data on *C. portucalensis* genomes were downloaded (Table S6) from the NCBI Reference Sequence (RefSeq) database (O'leary et al. 2016). PhyloPhlAn (v3.1.68) (Asnicar et al. 2020) was used to construct the phylogenetic tree of *Citrobacter portucalensis* MAG and public genomes based on sequence alignment.

### 2.6 Data Analysis

The linear discriminant analysis (LDA) effect size (LEfSe) was used to perform differential analysis of taxa. Principal coordinates analysis (PCoA) and permutational multivariate analysis of variance (PERMANOVA; response variables: Bray–Curtis dissimilarity; factors: groups; strata: locations; permutations = 999; method = “bray”) were used to evaluate the similarities and differences in the composition of the sample communities based on Bray–Curtis distances (Li et al. 2022). Multivariate homogeneity of group dispersions (variances) was assessed by using the betadisper in conjunction with a permutation test (*n* = 999 permutations, strata: locations) (Anderson and Walsh 2013). A Mantel test with 9999 permutations was conducted to evaluate the correlation between Bray–Curtis distance and geographic distance. The statistical analysis was performed by R (v4.3.0) using vegan (v2.6–4) and ggplot2 (v4.3.2) packages. The *p* values for α‐diversity indices (Shannon, Simpson, Chao1, and ACE), bacterial relative abundance, and the abundance of ARGs and VFs between groups were assessed using generalized linear mixed models (GLMMs) that incorporated sampling sites as random effects. Intergroup differences were subsequently evaluated through post hoc pairwise comparisons with Tukey's honestly significant difference (HSD) adjustment. Statistical analysis of metagenomic profiles (STAMP) (v2.1.3) (Parks et al. 2014) was used to predict the different functional pathways among the three groups using Welch's t‐test and Storey's false discovery rate (FDR) correction. The Spearman rank correlation analysis was used to analyze the correlation between ARGs and bacteria.

## 3 Results

### 3.1 Composition of Intestinal Microbiota of *Bufo gargarizans* in Different Habitats

**FIGURE 1:**
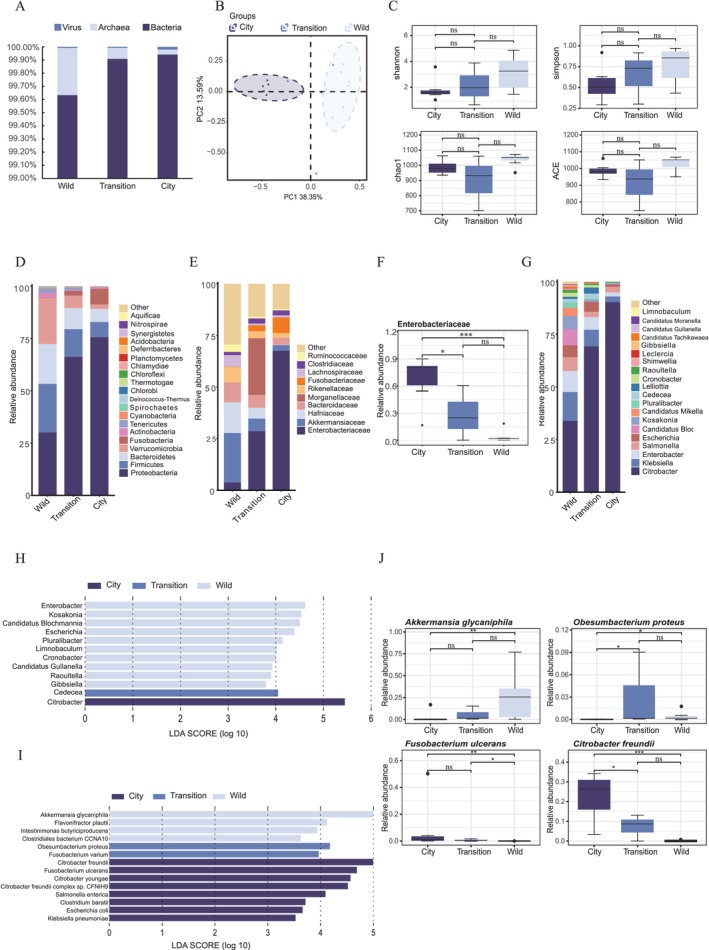
The comparisons of the intestinal microbiota composition of *Bufo gargarizans* in different habitats. (A) The proportion of bacteria, archaea, and viruses in the intestinal microbiota of the three groups. (B) Genus‐level β‐diversity among the three groups based on PCoA and PERMANOVA test of Bray–Curtis dissimilarity (*R* ^2^ = 0.368, *p* = 0.001). (C) The differences in α‐diversity in the three groups. (D) The top 20 bacteria by relative abundance at the phylum level across three groups. (E) The top 10 bacteria by relative abundance at the family level across three groups. (F) The comparison of the relative abundance of Enterobacteriaceae among the three groups. (G) The top 20 bacteria of Enterobacteriaceae at the genus level across three groups by relative abundance. (H) The different microbes at the genus level of Enterobacteriaceae among three groups by LEfSe (*p* < 0.05 and LDA > 2.0). (I) The different microbes at the species level among three groups by LEfSe (*p* < 0.05 and LDA > 3.5). (J) The comparison of the relative abundance of *Akkermansia glycaniphila*, *Obesumbacterium proteus* , *Fusobacterium ulcerans*, and *Citrobacter freundii* among the three groups. ^ns^ *p* > 0.05, *p < 0.05;**p < 0.01; ***p < 0.001 in Panels C, F, and J.

Metagenomic sequencing was carried out on 16 intestinal contents samples of *B. gargarizans* in three different habitats, resulting in 138.13 Gb of raw data and 136.41 Gb of filtered clean data. The average number of raw reads sequenced per sample was 57,549,955, and the average number of nonhost reads sequenced per sample was 52,935,011 (Tables S2 and S3). Based on metagenomic sequencing, the intestinal microbiota of the three groups was classified into 3 kingdoms, 40 phyla, 430 families, 1348 genera, and 4,395 species. Bacteria, archaea, and viruses account for 99.90%, 0.08%, and 0.02% of the total intestinal microbiota in the three groups. From the wild group to the city group, the proportion of bacteria and viruses increased while that of archaea decreased (Figure 1A). Results showed significant differences in the β‐diversity of the intestinal microbiota taxonomy among the wild, transition, and city groups (*R* ^2^ = 0.368, *p* = 0.001; Figure 1B). The permutation test for homogeneity of multivariate dispersions revealed no statistically significant differences in dispersion among groups (*p* > 0.05). The composition of microbial communities in the transition group was more similar to that in the wild group (*R* ^2^ = 0.169, *p* = 0.069) than that in the city group (*R* ^2^ = 0.247, *p* = 0.049). We found no significant spatial autocorrelation between microbial community dissimilarity and geographic distance. The Mantel test revealed a weak positive correlation (Mantel *R* = 0.083, *p* = 0.187), indicating that geographic distance explained less than 1% of the variation in community composition (*R* ^2^ = 0.007). In addition, there were no significant differences in diversity and richness of intestinal microbiota among the three groups based on α‐diversity (Figure 1C).

Since bacteria accounted for the vast majority of the intestinal microbiota in the three groups, we then focused on the comparison of the bacterial communities. At the phylum level, Proteobacteria (73.99%), Firmicutes (7.21%), Fusobacteria (6.90%), Bacteroidetes (5.78%), Verrucomicrobia (4.87%), and Actinobacteria (0.62%) exhibited a high relative abundance in the intestinal microbiota of *B. gargarizans* . Moreover, the relative abundance of Firmicutes, Bacteroidetes, Verrucomicrobia, and Actinobacteria decreased gradually while Proteobacteria and Fusobacteria increased gradually from the wild group to the city group (Figure 1D). At the family level, Ruminococcaceae (phylum Firmicutes, 0.62%), Akkermansiaceae (phylum Verrucomicrobia, 5.16%), Bacteroidaceae (phylum Bacteroidetes, 2.43%), and Rikenellaceae (phylum Bacteroidetes, 2.50%) gradually decreased in relative abundance from the wild group to the city group, while Enterobacteriaceae (phylum Proteobacteria, 53.76%) and Fusobacteriaceae (phylum Fusobacteria, 7.26%) showed a clear increase (Figure 1E). Specifically, Enterobacteriaceae occupied a significantly higher relative abundance in the city group (67.53%) compared to the other two groups (*p* < 0.05, Figure 1E,F).

Because of the obvious changes in Enterobacteriaceae among the three groups, a genus‐level Enterobacteriaceae profile was further explored. *Citrobacter* had the highest relative abundance among the three groups (47.27%) at the genus level (Figure 1G), especially in the city group (59.84%). The results of LEfSe analysis showed that significant differences were observed in 12 microbes at the genus level of Enterobacteriaceae among the three groups (LDA > 2.0). Among these, the relative abundances of *Enterobacter*, *Kosakonia*, *Candidatus Blochmannia*, *Escherichia*, *Pluralibacter*, *Limnobaculum*, *Cronobacter*, *Candidatus Gullanella*, *Raoultella*, and *Gibbsiella* were significantly higher in the wild group. *Cedecea* exhibited a significantly greater relative abundance in the transition group, while the relative abundance of *Citrobacter* was significantly increased in the city group (Figure 1H).

Next, we conducted the LEfSe at the species level of all bacterial communities to identify bacterial biomarkers in each group (LDA > 3.5) (Figure 1I). There were significant differences in 14 microbes. *Akkermansia glycaniphila*, *Flavonifractor plautii* , *Intestinimonas butyriciproducens*, and *Clostridiales bacterium CCNA10* were significantly increased in the wild group, while *Obesumbacterium proteus* and *Fusobacterium varium* were significantly increased in the transition group. In the city group, *Citrobacter freundii* , *Fusobacterium ulcerans* , *Citrobacter youngae* , *Citrobacter freundii complex* sp. *CFNIH9*, *Salmonella enterica* , *Clostridium baratii* , *Escherichia coli* , and *Klebsiella pneumoniae* showed significant increases.

Based on the above results, we set the LDA score > 4.9 for the city and wild groups and the LDA score > 4.0 for the transition group to find biomarkers with a greater influence in each group. We generated box plots to visualize differences in four bacterial biomarkers among the three groups. In the comparison between city and wild groups, the relative abundance of *A. glycaniphila* was remarkably higher in the wild group (*p* < 0.01) and the relative abundance of *C. freundii* was remarkably higher in the city group (*p* < 0.01). Compared to the city group, the relative abundance of *O. proteus* was significantly higher in the transition group (*p* < 0.05), as well as in the wild group (*p* < 0.05). Compared to the wild group, the relative abundances of *F. ulcerans* were significantly higher in the transition (*p* < 0.05) and city groups (*p* < 0.01) (Figure 1J).

### 3.2 The Functional Characterization of *Bufo gargarizans* in Different Habitats

**FIGURE 2:**
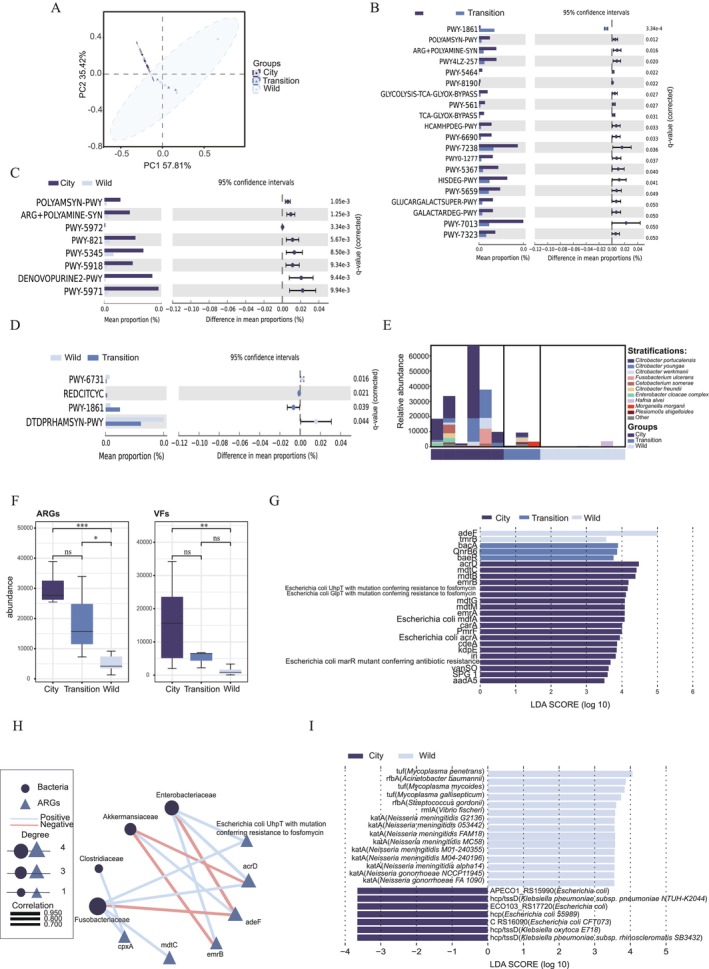
The comparisons of the intestinal microbiome function among the three groups. (A) β‐Diversity among the three groups based on PCoA and PERMANOVA test of Bray–Curtis dissimilarity (*R* ^2^ = 0.441, *p* = 0.001). (B) The different metabolic pathways between the city and transition group (*p* < 0.05). (C) The different metabolic pathways between the city and wild group (*p* < 0.01). (D) The different metabolic pathways between the wild and transition group (*p* < 0.05). (E) The metabolic pathway contributing bacteria of three groups. (F) The comparison of the abundance of ARGs and VFs among the three groups. (G) The different ARGs among the three groups by LEfSe (*p* < 0.05 and LDA > 3.5). (H) The Spearman rank correlation analysis between ARGs and bacteria (−0.7 < r_s_ < 0.7). (I) The different VFs among the three groups by LEfSe (*p* < 0.05 and LDA > 3.5). ^ns^ *p* > 0.05, *p < 0.05;**p < 0.01; ***p < 0.001 in Panel F.

According to the PERMANOVA test of Bray–Curtis dissimilarity, the microbial metabolic pathways of the intestinal microbiota in the three groups had significant differences (*R* ^2^ = 0.441, *p* = 0.001; Figure 2A). The permutation test for homogeneity of multivariate dispersions revealed no statistically significant differences in dispersion among groups (*p* > 0.05). The Mantel test also revealed a weak positive correlation (Mantel *R* = 0.093, *p* = 0.253), indicating that geographic distance explained less than 1% of the variation in pathways composition (*R* ^2^ = 0.009). Consistent with the composition of the microbial community, the pathways of microbial communities in the transition group were more similar to those in the wild group (*R* ^2^ = 0.037, *p* = 0.963) than to those in the city group (*R* ^2^ = 0.749, *p* = 0.007). We compared the groups in pairs to look for differential metabolic pathways. By comparing the city group with the wild group and the transition group, we found that the city group exhibited enrichment in a greater number of metabolic pathways than the other two groups. Moreover, the pathways associated with Enterobacteriaceae, like *E. coli* , *C. freundii* , *Klebsiella oxytoca* , *Salmonella enterica* , and *Yersinia pseudotuberculosis* , accounted for 60% of the enriched metabolic pathways in the city group. In particular, the pathways associated with *Escherichia coli* , such as the superpathway of polyamine biosynthesis I, superpathway of arginine and polyamine biosynthesis, and superpathway of heme *b* biosynthesis from glutamate, are highlighted (Table S4, Figure 2B,C). Moreover, the formaldehyde assimilation II (assimilatory RuMP cycle) and TCA cycle VI (*Helicobacter*) increased (*p* < 0.05) in the transition group, while the starch degradation III and dTDP‐β‐L‐rhamnose biosynthesis increased (*p* < 0.05) in the wild group (Figure 2B,D).

By analyzing the metabolic pathway‐contributing bacteria of each group, we found that Enterobacteriaceae were the largest group of bacteria contributing to all metabolic pathways after removal of unclassified bacteria in the three groups, accounting for 66.21% of the total abundance of pathway contributors. At the genus level, *Citrobacter* (59.89%) contributed the most to the metabolic pathways, particularly *C. portucalensis* (38.90%), and they were highly involved in the metabolic pathways of the city group and the transition group. In the metabolic pathway of the wild group, *Hafnia alvei* (4.32%) and *A. glycaniphila* (0.90%) contributed the most (Figure 2E). These results showed a similar trend to the taxonomic identification results.

Next, we predicted ARGs (antibiotic resistance genes) and VFs (virulence factors) of each group based on CARD and VFDB databases. A total of 845 ARGs, belonging to 235 AMR (antimicrobial resistance) gene families, and 2476 VFs across 13 categories were annotated. The types of ARGs related to resistance‐nodulation‐cell division (RND) antibiotic efflux pump and the types of VFs related to “Adherence” VF categories were the most in each group (Figure S2A,B). Among the three groups, the city group had the largest number of ARG types (*n* = 574) and unique ARGs (*n* = 332), as well as VFs. The transition group had the least number of ARG types (*n* = 257) and unique ARGs (*n* = 94) while the wild group had the least number of VF types (*n* = 570) and unique VFs (*n* = 72) (Figure S2C,D). Furthermore, the results indicated that the abundance of ARGs and VFs increased successively from the wild group to the city group, with significantly higher levels observed in the city group (Figure 2F).

Among the top 10 ARGs by abundance, the top four ARGs all belonged to resistance‐nodulation‐cell division (RND) antibiotic efflux pump and were associated with aminoglycoside antibiotics, aminocoumarin antibiotics, fluoroquinolone antibiotics, and tetracycline antibiotics (Figure S2E). In addition, *acrD*, *mdtC*, *mdtB*, *Escherichia coli UhpT with mutation conferring resistance to fosfomycin*, and *emrB* were the most abundant and significantly increased in the city group (LDA > 3.5) (Figure S2E,G). Moreover, we found that significant differences were observed in 25 ARGs among three groups, and all groups had differential ARGs associated with fluoroquinolone antibiotics. In addition, ARGs related to penam, phosphonic acid antibiotics, phenicol antibiotics, glycylcycline, rifamycin antibiotics, lincosamide antibiotics, macrolide antibiotics, cephalosporin, carbapenem, and triclosan were also obviously increased in the city group (Figure 2G). In the Spearman rank correlation analysis (Figure 2H), it was found that the number of ARGs positively correlated with Fusobacteriaceae and Enterobacteriaceae was the highest. All ARGs associated with Fusobacteriaceae and Enterobacteriaceae, except for *cpxA*, exhibited the highest abundance and significant enrichment in the city group among the three groups.

The top 10 VFs by abundance belonged to regulation, adherence, and effector delivery system categories. *Hcp* and *hcp/tssD* VFs were significantly increased in the city group (LDA > 3.5, *p* < 0.05) (Figure S2F,I), and they were related to Enterobacteriaceae bacteria. *TufA* VFs were most abundant in the transition group and were associated with *Francisella*, while *cdrA* was most abundant in the wild group and associated with *Legionella*. All of the VFs enriched in the city group belonged to the effector delivery system category, and all were related to Enterobacteriaceae (Figure 2I).

### 3.3 Reconstruction and Analysis of MAGs

Metagenomic binning of 16 intestinal metagenomes of *B. gargarizans* from our study was performed. After preliminarily completing metagenomic binning, we obtained 838 raw bins from intestinal metagenomes of *B. gargarizans* . Following the removal of redundant bins and setting completeness to > 70% and contamination to < 10%, we further obtained 322 nonredundant MAGs. Of these MAGs, 199 MAGs had completeness > 90% and contamination < 5%, which were defined as high‐quality draft genomes (Bowers et al. 2017). A total of 122 MAGs had completeness > 95% and contamination < 5%, and 14 MAGs had completeness > 97% and no contamination (Figure S2G and Table S5). Taxonomic labels of all MAGs were identified to order level at least. All 322 MAGs were identified to 2 kingdoms, 11 phyla, 16 classes, and 30 orders. Additionally, 318 MAGs were identified in 43 families, 229 MAGs in 79 genera, and 18 MAGs in 10 species (Table S5). At the kingdom level, there were 319 MAGs belonging to bacteria while 3 MAGs belonged to archaea.

**FIGURE 3:**
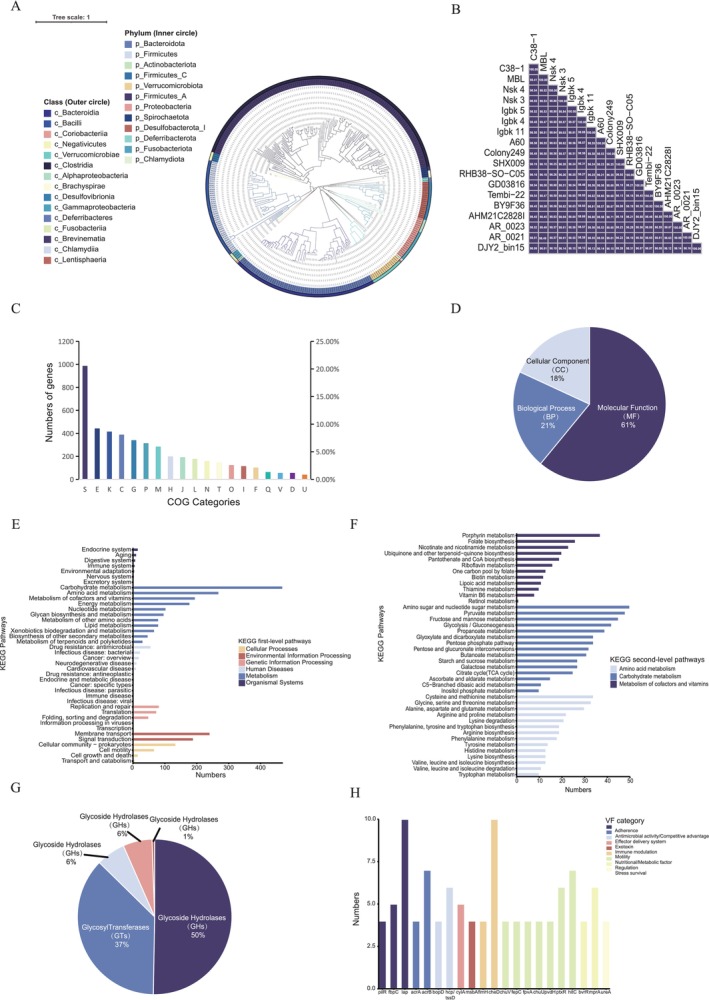
The analysis of MAGs. (A) The phylogenetic tree of 319 MAGs belonged to bacteria. (B) Comparison of ANI between the assembled *Citrobacter portucalensis* and 17 other strains. (C) COG functional classification of assembled *Citrobacter portucalensis*. (D) GO function of assembled *Citrobacter portucalensis*. (E) KEGG first‐level pathway classification of assembled *Citrobacter portucalensis*. (F) KEGG second‐level pathway classification of carbohydrate metabolism, amino acid metabolism, and the metabolism of cofactors and vitamins in assembled *Citrobacter portucalensis*. (G) The annotated results of carbohydrate‐active enzymes of *Citrobacter portucalensis*. (H) The top 21 VFs functional categories of assembled *Citrobacter portucalensis*.

The phylogenetic tree of the 319 MAGs belonging to bacteria was constructed and classified into 12 bacterial phyla. Most MAGs belonged to Firmicutes_A (class Clostridia, *n* = 134), followed by Bacteroidota (class Bacteroidia, *n* = 67), Firmicutes (class Bacilli, *n* = 46), Proteobacteria (class Gammaproteobacteria, *n* = 15; class Alphaproteobacteria, *n* = 6), and Desulfobacterota_I (class Desulfovibrionia, *n* = 18) (Figure 3A). All bins were also clustered at a threshold of 95% ANI to obtain 18 species‐level genome bins (SGBs). Therefore, these SGBs represented taxa of the 322 MAGs at the species level. They belonged to the family Enterobacteriaceae (*n* = 13), Fusobacteriales (*n* = 3), Clostridiales (*n* = 1), and Verrucomicrobiales (*n* = 1). Most of the SGBs belonged to *C. portucalensis* (*n* = 5) (Table S5). The MAGs that were not classified to species level (*n* = 304) might be novel species, belonging to 2 kingdoms, 16 classes of 13 phyla, and mainly including 133 MAGs of class Clostridia, 67 MAGs of class Bacteroidia, 46 MAGs of class Bacilli, 18 MAGs of class Desulfovibrionia, and 14 MAGs of class Verrucomicrobiae. The MAGs not classified to genus level (*n* = 93) mainly included 18 MAGs of order Erysipelotrichales, 16 MAGs of order Desulfovibrionales, 14 MAGs of order Oscillospirales, and 11 MAGs of order Lachnospirales (Table S5).

To further compare the functional features, we predicted and annotated the genes of these MAGs. We also predicted ARGs and VFs, and a total of 1205 ARGs and 1358 VFs were annotated. At the family level, more ARGs and VFs were annotated in Lachnospiraceae, Ruminococcaceae, Rikenellaceae, Enterobacteriaceae, Erysipelotrichaceae, Desulfovibrionaceae, and Aeromonadaceae (Figure S3A,B).

Based on all of the above analysis, we further analyzed the 13 SGBs belonging to Enterobacteriaceae. The city group had the most SGBs, the wild group had the least belonging to Enterobacteriaceae, and two SGBs from the wild group belonged to *H. alvei* while *C. portucalensis* dominated in the city and transition groups (Table S5).

Of these 13 SGBs, the one belonging to *C. portucalensis* with number DJY2_bin.15 annotated the most types of ARGs and VFs (Figure S3C). In addition, this MAG had a high degree of completeness and a low degree of contamination. Thus, we further analyzed this MAG. The genome size of *C. portucalensis* DJY2_bin.15 was 5,125,049 bp with a GC content of 51.64%. The number of predicted genes was 3866, and 6 rRNAs, 69 tRNAs, and 1 tmRNA were present. We downloaded all *C. portucalensis* data from the RefSeq database, 243 in total (Table S6). Then, the average nucleotide identity (ANI) between the assembled *C. portucalensis* and all the public genome data were calculated. After constructing the phylogenetic tree, the 17 most closely related species were selected (Figure S3D). The assembled *C. portucalensis* was most closely related to species numbered SHX009 (ANI = 98.28) and RHB38‐SO‐C05 (ANI = 98.33) (Figure 3B).

In function annotation, genes associated with molecular function as well as amino acid transport and metabolism were predominantly identified (Figure 3C,D). Besides, six KEGG first‐level pathways, 41 second‐level pathways, and 243 third‐level pathways were annotated based on the KEGG database. The annotated genes related to metabolism were the most abundant (Figure 3E). The abundance of genes associated with carbohydrate metabolism, amino acid metabolism, and metabolism of cofactors and vitamins was highest in metabolic pathways. Among these three second‐level pathways, amino sugar and nucleotide sugar metabolism, cysteine and methionine metabolism, and porphyrin metabolism had the highest abundance of related genes, respectively (Figure 3F). Annotation results in the CAZy database revealed that GHs and GTs were the predominant carbohydrate‐active enzymes (Figure 3G), accounting for 16 of the top 20 most abundant enzymes. Finally, a total of 230 types of ARGs belonged to 76 AMR gene families, and 367 types of VFs belonged to 14 VF categories were annotated. The largest number of ARGs were related to the resistance‐nodulation‐cell division (RND) antibiotic efflux pump. *MacB*, *adeL*, and *NmcR* were the most abundant ARGs, and they were associated with tetracycline, erythromycin, and beta‐lactam antibiotics (cefoxitin, cefazolin, and ampicillin) (Table S7). The largest number of virulence genes is related to immune modulation. *cheD* and *lap* were the most abundant VFs, and they were associated with motility and adherence, which might help the bacteria cause disease (Figure 3H).

## 4 Discussion

Our study found that the structure and function of the intestinal microbiota of *B. gargarizans* were significantly different in different habitats. The wild group was mainly composed of Akkermansiaceae, Hafniaceae, and Bacteroidaceae, with *A. glycaniphila* and *F. plautii* significantly enriched. Studies have shown that *A. glycaniphila* has a close phylogenetic relationship with *Akkermansia muciniphila* (Ouwerkerk et al. 2016), which is known to have great probiotic potential in regulating hosts' intestinal microbiota and ameliorating disease (Ding et al. 2021; Grajeda‐Iglesias et al. 2021; Xie et al. 2023). *Flavonifractor plautii* has been reported as having a positive effect in reducing inflammation in obese adipose tissue and protecting against elevated arterial stiffness (Luo et al. 2023; Mikami et al. 2020). These bacteria may play an important role in regulating the normal physiological functions of the Asiatic toad and in preventing diseases.

In our study, Enterobacteriaceae showed significant changes in the three habitats and occupied a high relative abundance in the city group, especially for *Citrobacter*. They may cause diseases in toads and other amphibians, including *C. freundii* (Latney and Klaphake 2013; Wang and Xie 2024). *Citrobacter*, a conditional pathogen, is found in sewage, human and animal feces, food, and other environments (Ekwanzala et al. 2020; Liu et al. 2017; Phuadraksa et al. 2022). The enrichment of Enterobacteriaceae in the city group is likely driven by human activities (Díaz et al. 2024). These facilitate the proliferation of Enterobacteriaceae, such as *Citrobacter*, in the living environment of *B. gargarizans* , particularly in aquatic habitats. Besides, the intestinal microbiota structure of the transition groups showed a transition state between the city group and the wild group. Therefore, our findings suggested that the intestinal microbiota of *B. gargarizans* could reflect the effects of human activities on the environment.

Urban activities enhance the diversity of ARGs and VFs in animals (Zhao et al. 2022); we also found that the abundance and diversity of ARGs and VFs increased as human activities rose. Urban human activities are important sources of ARGs and VFs accumulation, such as the use of plastics, food garbage, sewage discharge, and heavy use of antibiotics (Cabello 2006; Hendriksen et al. 2019; Kanger et al. 2020; Yang et al. 2019). Thus, this may increase the likelihood of transmission to *B. gargarizans* living in urban areas, causing ARGs and VFs to spread in their animals. This serves as a reminder for urban environmental management.

In addition, we found that the ARGs and VFs in the city group were highly correlated with Enterobacteriaceae, which are likely effective carriers of these genes. However, Enterobacteriaceae have often been reported to be resistant to beta‐lactam antibiotics (Iredell et al. 2016). Of the 410 ARGs annotated in 13 Enterobacteriaceae we assembled, 93 ARGs were associated with beta‐lactam antibiotics (22.68%) (Table S8). Beta‐lactamase enzymes are the primary resistance mechanism to beta‐lactam antibiotics (Tooke et al. 2019). Therefore, we did a further count and found that 30 ARGs produce beta‐lactamase, which causes antibiotic inactivation; especially, *NmcR* resistance genes have the highest abundance. In the MAG named *C. portucalensis* DJY2_bin.15 that we filtered out, we identified 53 types of ARGs (23.14%) associated with six beta‐lactam antibiotics, of which nine conferred resistance to beta‐lactam antibiotics by producing beta‐lactamase (Table S7). *NmcR* also had the most abundant among them. Besides, *C. portucalensis* is a global multidrug‐resistant (MDR) pathogen that is resistant to beta‐lactam antibiotics, especially carbapenems (Sellera et al. 2022). In our study, we found that among the 59 ARGs associated with beta‐lactam antibiotics in *C. portucalensis* DJY2_bin.15, 49 ARGs were associated with carbapenems (83.05%). These genes are present but not necessarily expressed. Without metatranscriptomic, metaproteomic, or phenotypic validation data, these bacteria may not necessarily express antibiotic resistance. However, the presence of ARGs increases the risk of their spread within or among Asiatic toads (Zhuang et al. 2024).

We were also concerned about the previous studies on *C. portucalensis* and diseases. Notably, the overrepresentation of Enterobacteriaceae, including *C. portucalensis*, is a key gut microbiome signature in inflammatory bowel diseases (IBD) (Khorsand et al. 2022). Studies have shown that IBD is characterized by dysregulation of key metabolic pathways—including amino acid metabolic pathways, the tricarboxylic acid (TCA) cycle, and pyrimidine biosynthesis—alongside abnormal levels of associated metabolites (Connors et al. 2018; Lavelle and Sokol 2020; Morgan et al. 2012; Qiu et al. 2022). Especially, the increase in tryptophan metabolism is related to intestinal inflammation, and the lack of tryptophan may lead to the development of IBD or increase in disease activities (Bajaj et al. 2024; Nikolaus et al. 2017). These metabolic pathways were annotated in *C. portucalensis* in our study, and they may be involved in the progression of intestinal inflammation through these pathways. However, we still do not know whether this bacterium will cause diseases such as enteritis in amphibians, similar to those caused by *C. freundii* . After that, we can further investigate its influence on the gastrointestinal system and its role in the occurrence and progression of intestinal inflammation in the Asiatic toad through cellular experiments, animal studies, and metabolomics.

Our study provided valuable insights into the characteristics of the intestinal microbiota in different habitats of *B. gargarizans*. Our work suggested that the intestinal microbiota of *B. gargarizans* could be used as one of the environmental indicators to reflect environmental pollution and changes. Furthermore, the accumulation of Enterobacteriaceae, particularly *Citrobacter*, in the intestines of *B. gargarizans* in city areas is noteworthy. We proposed that the *Citrobacter* in the intestinal microbiota of *B. gargarizans* could serve as a primary indicator for reflecting environmental changes and human activities. However, in this study, we did not analyze the relationship between environmental samples and intestinal microbiota, which should be further discussed in combination with environmental samples. Our future research will focus on the association analysis between environmental samples and the intestinal microbiota of *B. gargarizans*. Furthermore, considering the important role of *C. portucalensis* in intestinal diseases and the lack of research on its role in amphibians, we suggested investigating the effects of this bacterium on the intestinal health of the Asiatic toad to raise awareness about the health of this species.

## Author Contributions

**Xuena Kang:** data curation (equal), formal analysis (equal), investigation (equal), visualization (equal), writing – original draft (lead). **Meiying Shao:** investigation (equal), methodology (equal), software (equal), writing – review and editing (equal). **Jiyang Jiang:** formal analysis (equal), software (equal), validation (equal), writing – review and editing (equal). **Lewei He:** formal analysis (equal), validation (equal). **Yunwei Lu:** methodology (equal), writing – review and editing (equal). **Jiarong Song:** writing – review and editing (equal). **Jue Xu:** resources (equal), writing – review and editing (equal). **Zhenxin Fan:** funding acquisition (equal), project administration (lead), supervision (equal), writing – review and editing (equal).

## Conflicts of Interest

The authors declare no conflicts of interest.

## Supporting information


**Figure S1.** Sampling location map (Data sources included the 2022 land cover dataset in China (Jie and Xin 2021) and contemporaneous population density data from the sampling period (Sims et al. 2022; Sims et al. 2023).

**Figure S2.** The analysis of intestinal microbiota of *Bufo gargarizans* based on metagenomes and MAGs. (A) The AMR gene families of predicted ARGs among the three groups. (B) The VF categories of predicted VFs among the three groups. (C) The types of ARGs among the three groups. (D) The types of VFs among the three groups. (E) The top 10 most abundant ARGs in three groups. (F) The top 10 most abundant VFs in three groups. (G) The completeness and contamination of original MAGs.

**Figure S3.** The phylogenetic relationships and functions of assembled *Citrobacter portucalensis*. (A) The top 10 MAGs that annotated most types of ARGs at family level. (B) The top 10 MAGs that annotated most types of VFs at family level. (C) Numbers of ARGs and VF types in 13 MAGs belonging to Enterobacteriaceae. (D) Phylogenetic tree of assembled *C. portucalensis* and other 17 strains.

**Table S1.** The summary of information of *Bufo gargarizans* .
**Table S2.** Quality control data of metagenomic sequencing for all samples in this study.
**Table S3.** The information of metagenomic data after quality control.
**Table S4.** Detailed description of the functional pathway.
**Table S5.** The information on MAGs.
**Table S6.** Public genomic data of *Citrobacter portucalensis* based on RefSeq.
**Table S7.** The ARGs of MAG numbered DJY2_bin.15 (*Citrobacter portucalensis*).
**Table S8.** The ARGs associated with beta‐lactam antibiotics of 13 MAGs belonged to Enterobacteriaceae.

## Data Availability

The raw data of metagenomes have been submitted to the China National GeneBank DataBase (https://db.cngb.org/) with the accession number CNP0005874.
