# Supplementary figures and images for: The Gut Microbiome of the Asiatic Toad ( Bufo gargarizans ) Reflects Environmental Changes and Human Activities
Source: Ecol Evol. 2025 May 7;15(5):e71394. doi: 10.1002/ece3.71394 (PMC12058643; doi:10.1002/ece3.71394)

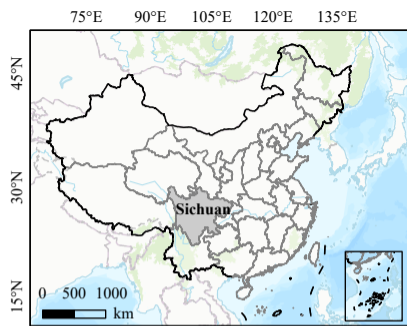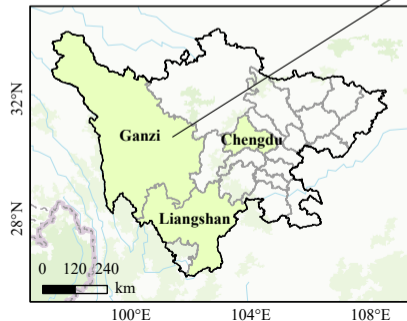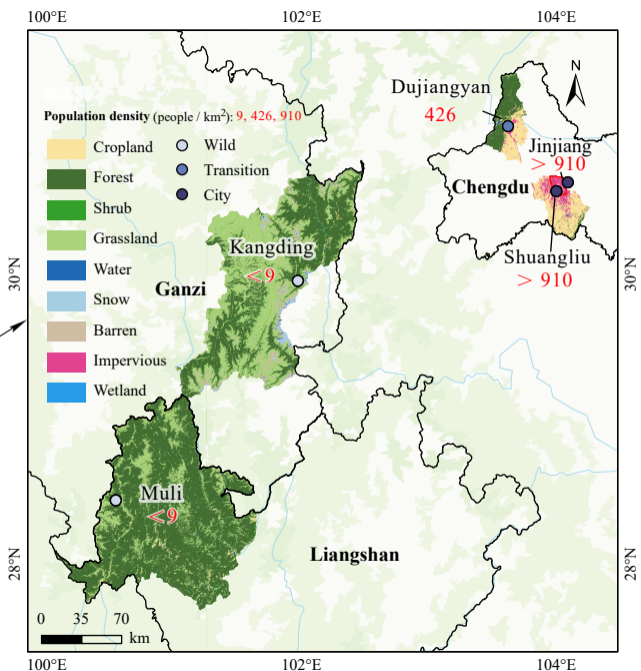

Supplement: Supplementary file 1 — Figure S1. Sampling location map (Data sources included the 2022 land cover dataset in China (Jie and Xin 2021) and contemporaneous population density data from the sampling period (Sims et al. 2022; Sims et al. 2023). [file ECE3-15-e71394-s001.pdf]

A

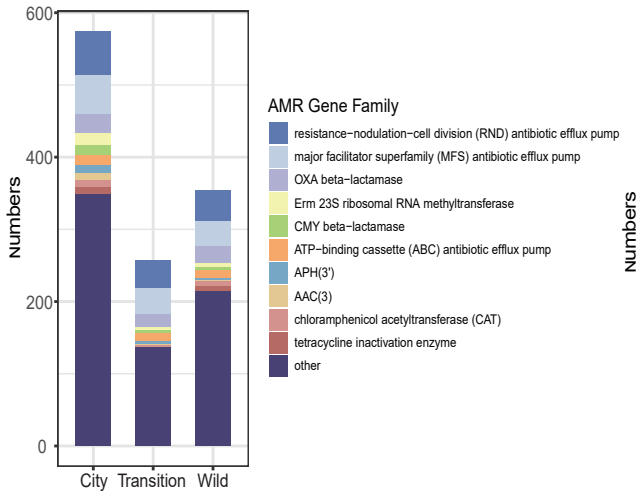

B

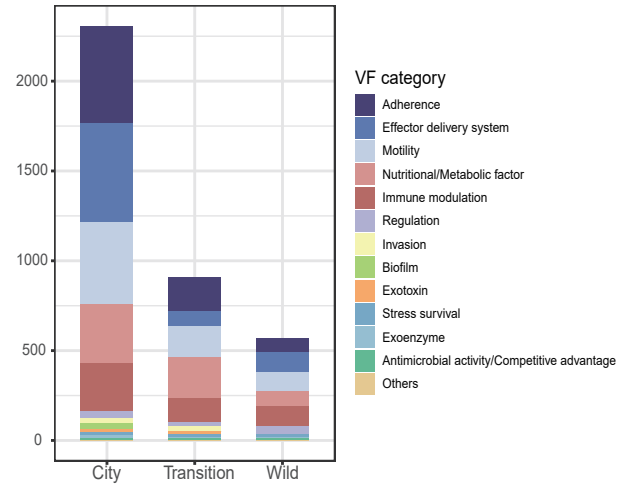

C

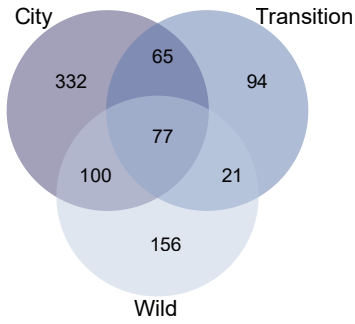

D

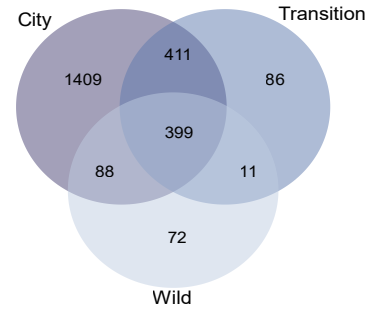

E

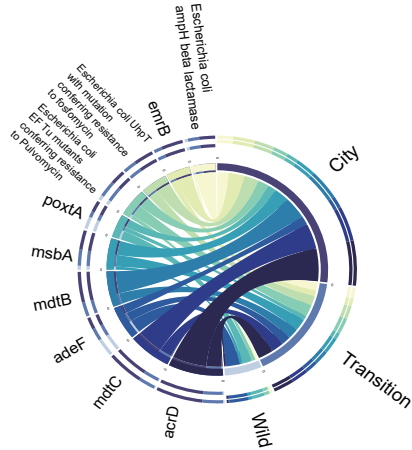

F

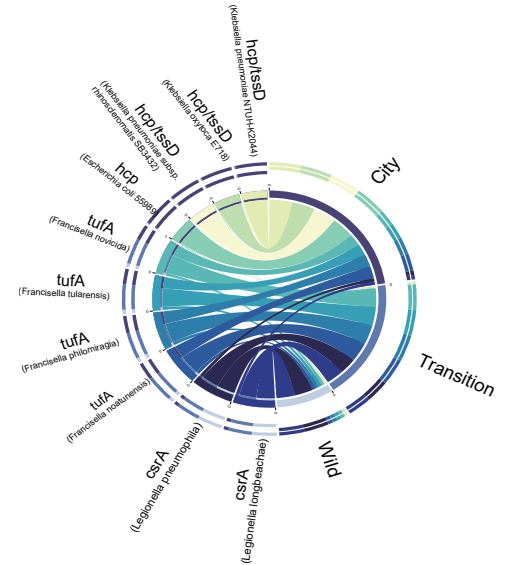

G

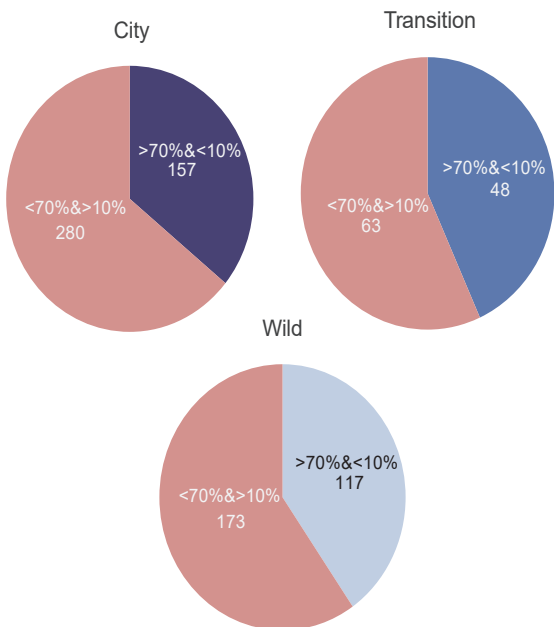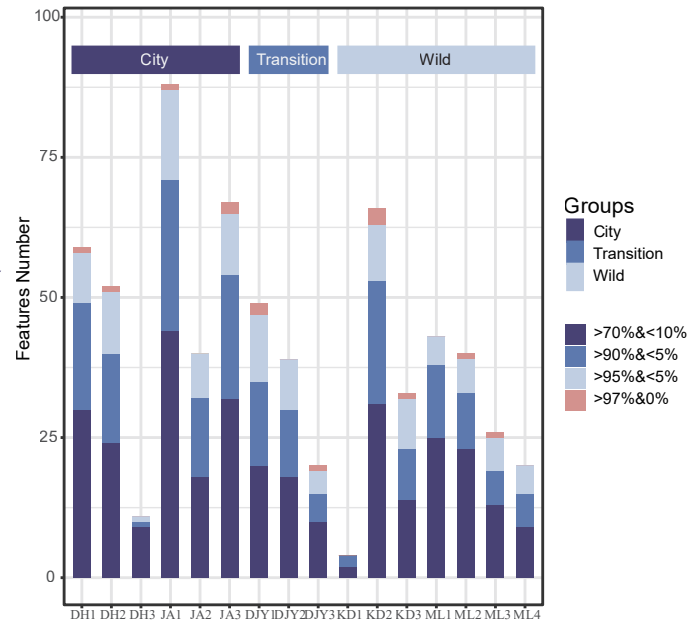

Supplement: Supplementary file 2 — Figure S2. The analysis of intestinal microbiota of Bufo gargarizans based on metagenomes and MAGs. (A) The AMR gene families of predicted ARGs among the three groups. (B) The VF categories of predicted VFs among the three groups. (C) The types of ARGs among the three groups. (D) The types of VFs among the three groups. (E) The top 10 most abundant ARGs in three groups. (F) The top 10 most abundant VFs in three groups. (G) The completeness and contamination of original MAGs. [file ECE3-15-e71394-s003.pdf]

A

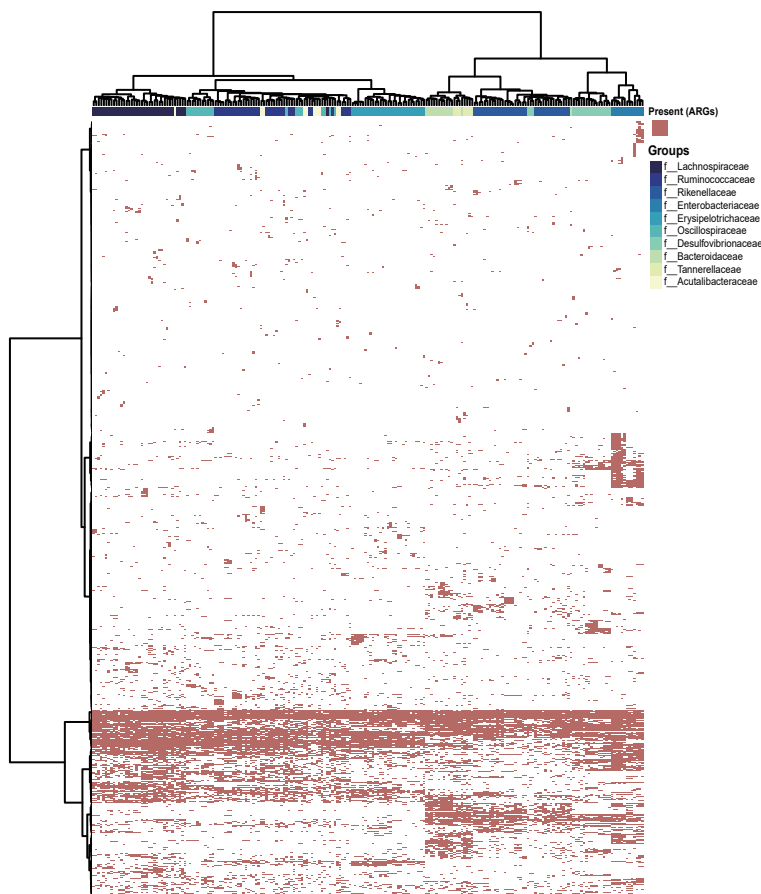

B

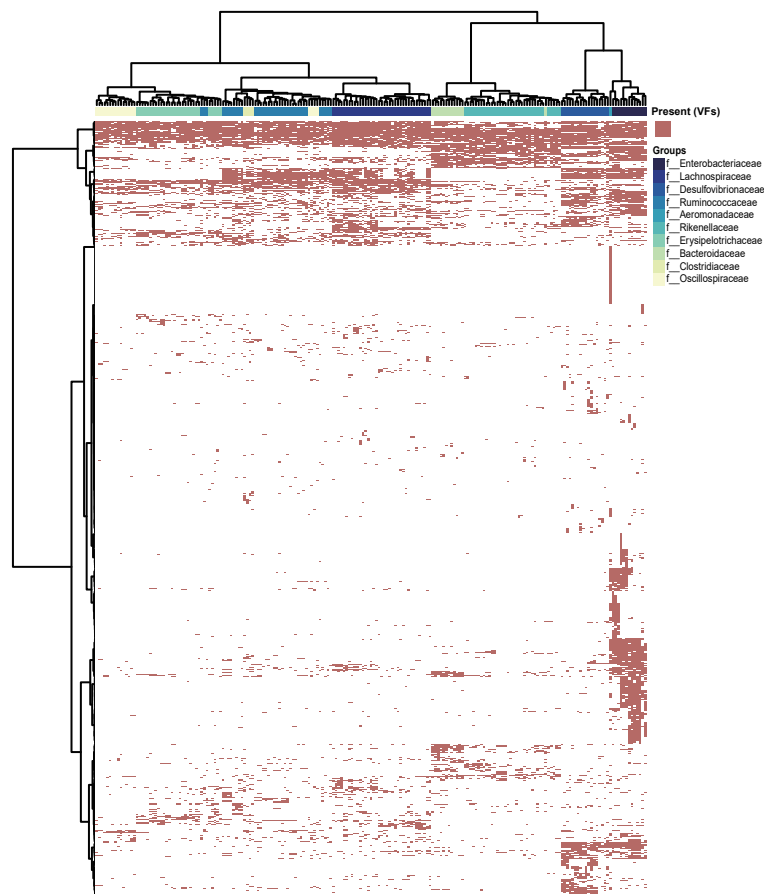

C

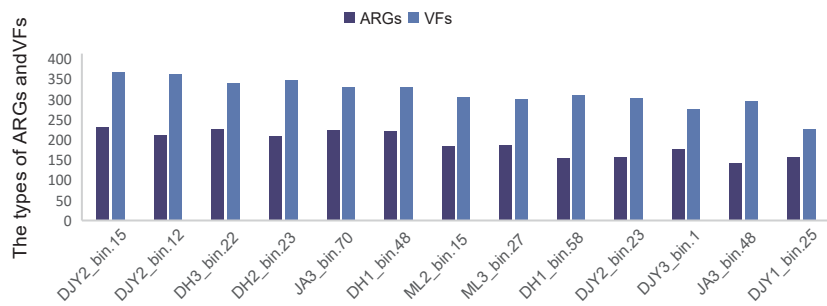

D

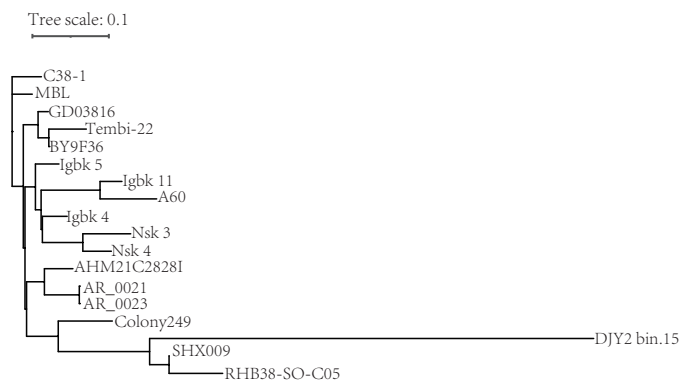

Supplement: Supplementary file 3 — Figure S3. The phylogenetic relationships and functions of assembled Citrobacter portucalensis. (A) The top 10 MAGs that annotated most types of ARGs at family level. (B) The top 10 MAGs that annotated most types of VFs at family level. (C) Numbers of ARGs and VF types in 13 MAGs belonging to Enterobacteriaceae. (D) Phylogenetic tree of assembled C. portucalensis and other 17 strains. [file ECE3-15-e71394-s004.pdf]
